# Variants c.677 C>T, c.1298 A>C in *MTHFR*, and c.66 A>G in *MTRR* Affect the Occurrence of Recurrent Pregnancy Loss in Chinese Women

**DOI:** 10.1089/gtmb.2020.0106

**Published:** 2020-11-10

**Authors:** Yan Zhang, Wenli Zhan, Qianyi Du, Li Wu, Hongke Ding, Fenghua Liu, Aihua Yin

**Affiliations:** ^1^ Medical Genetics Center, Guangdong Women and Children Hospital, Guangzhou, P.R. China.; ^2^ Maternal and Children Metabolic-Genetic Disease Key Laboratory of Guangdong Women and Children Hospital, Guangzhou, P.R. China.; ^3^ Health Care Institute, Guangdong Women and Children Hospital, Guangzhou, P.R. China.; ^4^ Reproductive Center, Guangdong Women and Children Hospital, Guangzhou, P.R. China.

**Keywords:** recurrent pregnancy loss, methylenetetrahydrofolate reductase, methionine synthase reductase, Southeast Chinese population

## Abstract

***Objective:*** Although genetic variants of key enzymes in the folic acid-methionine metabolic circulation, including methylenetetrahydrofolate reductase (MTHFR) and methionine synthase reductase (MTRR) were thought to be related to the risk of recurrent pregnancy loss (RPL), the results of recent studies have been inconsistent. Therefore, the present retrospective case–control study was designed to explore whether the variants c.66A>G in *MTRR* and c.677C>T and c.1298A>C in *MTHFR* are associated with the susceptibility of RPL in Southeast Chinese women.

***Materials and Methods:*** In total, samples from 237 RPL patients and 618 healthy controls were collected and genotyped by fluorescent quantitative polymerase chain reaction. The frequencies of the variants were calculated and compared between the two groups. The relative risk of the various genotypes was further determined by calculating the odds ratio (OR) at a 95% confidence interval (CI).

***Results:*** A significant positive correlation was observed between the variants *MTHFR* c.677C>T, *MTHFR* c.1298A>C, *MTRR* c.66A>G, and RPL susceptibility (*MTHFR* c.677C>T, OR = 0.74, 95% CI = 0.58–0.95, *p* = 0.02; *MTHFR* c.1298A>C, OR = 1.39, 95% CI = 1.09–1.77, *p* = 0.008; *MTRR* c.66A>G, OR = 1.38, 95% CI = 1.10–1.73, *p* = 0.006). Further analysis of the genotypic distributions of the three variants between the two groups showed that the *MTHFR* c.677C>T heterozygote was associated with lower RPL risk, while the *MTHFR* c.1298A>C variant and MTRR c.66A>G heterozygote were correlated with higher RPL risk (dominant model, *MTHFR* c.677C>T, OR = 0.70, 95% CI = 0.52–0.95, *p* = 0.02; *MTHFR* c.1298A>C, OR = 1.39, 95% CI = 1.03–1.88, *p* = 0.032; *MTRR* c.66A>G, OR = 1.62, 95% CI = 1.20–2.19, *p* = 0.002).

***Conclusion:*** *MTHFR* c.677C>T and c.1298A>C and *MTRR* c.66A>G were associated with RPL in Southeast Chinese women.

## Introduction

Recurrent pregnancy loss (RPL) is defined as two or more consecutive clinical pregnancy losses before the 20 completed weeks of gestation and is a significant clinical problem that affects up to 5% of women during their reproductive years and leads to both emotional and physical morbidity (Samantha *et al.*, 2013; Abu-Heija, 2014). Thus far, the etiology of RPL is still unclear. Although several etiological factors for RPL have been proposed, including anatomic abnormalities of the genital tract, cytogenetic abnormalities, and genetic and environmental factors, the underlying pathophysiological mechanisms for RPL remain unknown in ∼50% of cases (Larsen *et al.*, 2013; Puri *et al.*, 2013).

In recent years, research on the factors affecting RPL of unknown origin has focused on the disorder of folate-methionine metabolism and circulation, especially the gene variation of the key enzymes of this circulation, methylenetetrahydrofolate reductase (MTHFR), and methionine synthase reductase (MTRR). In the folic acid metabolic pathway, MTHFR catalyzes the conversion of 5,10-methylenetetrahydrofolate to 5-methylenetetrahydrofolate, which provides one-carbon unit to homocysteine (HCY). Once combined with methionine synthase (MTR), vitamin B12, and MTRR (which can maintain MTR activity), HCY can be methylated to methionine.

Several studies have shown that variants in *MTHFR* and *MTRR* have a relationship with reduced enzyme activity, which may lead to the disorder in the conversion of 5,10-methylenetetrahydrofolate to 5-methylenetetrahydrofolate and dysfunction in the methylation of HCY methionine (Mao *et al.*, 2010; Saravelos and Regan, 2014). Because 5-methylenetetrahydrofolate is an important substrate of the active folate acid, active folate acid and HCY might not be properly converted, which may result in lower active folate levels and higher HCY levels.

However, it is well known that folate acid is a necessity in many biological reactions, including DNA synthesis, cell growth, and cell division, especially in fetal development during pregnancy (Sharfstein, 2001; Dasarathy *et al.*, 2010). Thus, due to reduced folic acid levels, fetal DNA and protein synthesis may be reduced and may lead to spontaneous abortion. In addition, increased HCY levels have been shown to cause damage to vascular endothelial cells, body coagulation and the fibrinolytic system, and abnormal lipid metabolism. All of these factors, along with the placental toxicity of HCY and other mechanisms, contribute to spontaneous abortion (Obwegeser *et al.*, 1999; Poddar *et al.*, 2001).

Among the variants of *MTHFR*, c.677C>T and c.1298A>C are the most prevalent (Daher *et al.*, 2012; Kosova *et al.*, 2015). To date, several studies have explored the association between these two variants and RPL. However, their results are controversial. Some studies showed that *MTHFR* c.677C>T and c.1298A>C variants contribute to the risk of RPL, while others reported that there was no significant association between *MTHFR* variants and RPL (Dilley *et al.*, 2002; Goodman *et al.*, 2006; Jeddi-Tehrani *et al.*, 2011). c.66A>G is the most common variant in *MTRR* (Yousefian *et al.*, 2014; Ling *et al.*, 2018). Even though MTRR plays a crucial role in maintaining the activation state of MTR, few studies have explored the relationship between polymorphism c.66A>G in *MTRR* and RPL.

Therefore, to determine whether the polymorphism c.66A>G in the *MTRR* and *MTHFR* variants c.677C>T and c.1298A>C are associated with RPL, the present retrospective case–control study was conducted in a Southeast Chinese population.

## Materials and Methods

### Subjects

A total of 855 Chinese women were enrolled in a retrospective case–control study, including 237 RPL patients and 618 healthy controls from Guangdong Women and Children's Hospital. Recruited RPL patients met the inclusion criteria as follows: (1) diagnosed with RPL in compliance with the American Society for Reproductive Medicine definitions of infertility and recurrent pregnancy loss (Practice Committee of the American Society for Reproductive Medicine, 2013). (2) No anatomic disorders, endocrine dysfunction, antiphospholipid antibodies, or an abnormal karyotype. (3) None of the subjects had pregnancy-related complications, such as hypertension, thyroid abnormalities, or diabetes. The health controls included 618 healthy women with no history of miscarriages. The age of all the subjects ranged from 17 to 42 years. The characteristics of the case and control subjects, including the number of miscarriages, are presented in Table 1 and Supplementary Table S1.

**Table 1.** Characteristics of Subjects with Recurrent Pregnancy Loss and Controls

	RPL cases (*n* = 237)		Controls (*n* = 618)		p
	N	%	n	%	
Age (years)	237		618		0.675
17–29	158	66.67	461	74.60	
30–39	75	31.65	153	24.76	
≥40	4	1.69	4	0.65	
No. of miscarriages					
2	187	78.90	—	—	
3	44	18.57	—	—	
≥4	6	2.53	—	—	

### Ethics statement

This study was approved by the Ethics Committee at the Guangdong Women and Children Hospital and conducted in accordance with the 1975 Declaration of Helsinki. All the participants provided written informed consent to participate in this study and consented to sample collection.

### DNA extraction and genotyping

Peripheral venous blood samples were collected from the subjects with informed consent. Genomic DNA was extracted from blood using a DNA purification kit (QIAGEN, USA). Then, each polymorphism was genotyped by fluorescent quantitative polymerase chain reaction. The sequences of the primers and probes used are shown in Table 2. Genotyping was conducted in a 10 μL polymerase chain reaction (PCR) system that contained 1 μL of genomic DNA (2 ng/μL), 5 μL 2 × TaqMan universal master mix (TaKaRa, Japan), 0.5 μL 20 × TaqMan-MGB probes (Sangon, China), and 3.5 μL deionized water. The PCR conditions were as follows: 10 min at 95°C followed by 20 cycles at 92°C for 15 s, 60°C for 1 min, and 30 cycles at 89°C for 15 s, and 60°C for 90 s. The endpoint fluorescence was read by the ABI PRISM 7900HT sequence detection system, and the results were further analyzed following the manufacturer's instructions.

**Table 2.** The Primer and Probe Sequences Used in the Study

Variants site	Forward primer	Reverse primer	Vic-labeled MGB probe	Fam-labeled MGB probe
*MTHFR* c.677C>T	GAAAAGCTGCGTGATGATG	TTGAAGGAGAAGGTGTC	AATCG[G]CTCCCGC	AATCG[A]CTCCCGC
*MTHFR* c.1298A>C	AAGAACGAAGACTTCAAA	TGGGGGGAGGAGCTGAC	ACACTT[G]CTTCACT	ACACTT[T]CTTCACT
*MTRR* c.66A>G	AGGCAAAGGCCATCGCA	ATCCATGTACCACAGCTT	AAGAAAT[A]TGTGAG	AAGAAAT[G]TGTGAG

MGB, minor groove binder; MTHFR, methylenetetrahydrofolate reductase; MTRR, methionine synthase reductase.

### Statistical analysis

The allele and genotype frequencies of MTHFR and MTRR gene case–control comparisons were evaluated by chi-square (*χ*^2^) test or Fisher's exact test. The relative risk of the variant genotypes was determined by calculating the odds ratio (OR) at a 95% confidence interval (CI). Logistic regression analysis was performed. The linkage disequilibrium (LD) among the three variants was evaluated by Haploview 4.1, which refers to the nonindependence of alleles at different sites (Pritchard and Przeworski, 2001). A two-sided *p-*value <0.05 was considered statistically significant in all analyses. All statistical analyses were conducted using PLINK version 7.02.

## Results

### Characteristics of the included subjects

Part of the baseline characteristics of the included subjects is shown in Table 1. A total of 237 RPL cases and 618 healthy controls participated in the study (Supplementary Table S1). The age of the subjects from the two groups was matched (*p* = 0.675). The average age of RPL patients and controls was 27.85 and 27.01 years, respectively. Regarding the frequency of miscarriage, most of the RPL group had two miscarriages.

### Genotyping and LD evaluation

To explore the association between *MTHFR* and *MTRR* variants and the risk of RPL, real-time fluorescence PCR was used to genotype all the participants for c.677C>T, c.1298A>C in *MTHFR* and c.66A>G in *MTRR*. The veracity of the genotyping results was confirmed by random direct sequencing of PCR products. The sequencing results were all in concordance with the genotyping results. All genotypes were consistent with the Hardy–Weinberg equilibrium, which was determined at the 0.05 significance level.

LD evaluation was conducted via Haploview, which showed. *MTHFR* c.677C>T (rs1801133) and c.1298A>C (rs1801131) were in LD. *MTRR* c.66A>G (rs7870860) was in slight LD with either *MTHFR* c.677C>T (rs1801133) or c.1298A>C (rs1801131).

### Association analysis of *MTHFR* and *MTRR* variants with RPL susceptibility

Significant differences were observed between the RPL group and healthy controls in the allele distributions of the *MTHFR* variants c.677C>T and c.1298A>C and the *MTRR* c.66A>G (Table 3). The frequency of the T allele at c.677 in *MTHFR* (c.677C>T) in the control group was significantly higher than that of the RPL group (28.16% vs. 22.57%), which showed *MTHFR* (c.677C>T) allele T associated with lower RPL susceptibility (OR = 0.74, 95% CI = 0.58–0.95, *p* = 0.02). However, the frequencies of the C allele at c.1298 of *MTHFR* and the G allele at c.66 of *MTRR* in the healthy group were significantly lower than those in the RPL group (healthy group vs. RPL group: *MTHFR* c.1298A>C, 21.60% vs. 27.64%; *MTRR* c.66A>G, 26.62% vs. 33.33%, respectively), which showed that *MTHFR* (c.1298A>C) allele C and *MTRR* (c.66A>G) allele G were related to higher RPL susceptibility (*MTHFR* c.1298A>C, OR = 1.39, 95% CI = 1.09–1.77, *p* = 0.008; *MTRR* c.66A>G, OR = 1.38, 95% CI = 1.10–1.73, *p* = 0.006).

**Table 3.** *MTHFR* and *MTRR* Allele Frequencies for the Control and Recurrent Pregnancy Loss Groups

Allele	Observed frequencies		Age-adjusted OR (95% CI)	p^a^
	Cases (*n* = 237),* n *(%)	Controls (*n* = 618),* n *(%)		
*MTHFR* c.677C>T				
C	367 (77.43)	888 (71.84)	0.74 (0.58–0.95)	***0.02***
T	107 (22.57)	348 (28.16)		
*MTHFR* c.1298A>C				
A	343 (72.36)	969 (78.40)	1.39 (1.09–1.77)	***0.008***
C	131 (27.64)	267 (21.60)		
*MTRR* c.66A>G				
A	316 (66.67)	907 (73.38)	1.38 (1.10–1.73)	***0.006***
G	158 (33.33)	329 (26.62)		

^a^ Logistic regression analysis and the chi-square test were used to evaluate the data with 95% CIs. Significant findings are shown in bold-italic font.

CI, confidence interval.

Then, the genotypic distribution of these three variants between the two groups was compared. As shown in Table 4, the *MTHFR* c.677C>T heterozygote was associated with lower RPL susceptibility (OR = 0.72, 95% CI = 0.525–0.987, *p* = 0.041). This was supported by a further dominant model of T allele, including CT heterozygote or TT homozygote, was also associated with lower RPL susceptibility (OR = 0.699, 95% CI = 0.516–0.946, *p* = 0.020). For c.1298A>C, only its dominant model was associated with higher RPL susceptibility (OR = 1.392, 95% CI = 1.029–1.884, *p* = 0.032). The *MTRR* c.66A>G heterozygote was associated with higher RPL susceptibility (OR = 0.1645, 95% CI = 1.198–2.257, *p* = 0.002; dominant model, OR = 1.619, 95% CI = 1.197–2.190, *p* = 0.002).

**Table 4.** Individual *MTHFR* and *MTRR* Genotype Distributions for Spontaneous Abortion and Control Groups

Gene	Genotype frequencies		Age-adjusted OR	95% CI	p^a^
	Cases (*n* = 237),* n *(%)	Controls (*n* = 618),* n *(%)			
*MTHFR* c.677C>T					
CC	141 (59.49)	313 (50.65)	1		
CT	85 (35.86)	262 (42.39)	0.72	0.525–0.987	***0.041***
TT	11 (4.65)	43 (6.96)	0.568	0.284–1.134	0.105
CT+TT	96 (40.51)	305 (49.35)	0.699	0.516–0.946	***0.020***
CC+CT	226 (95.35)	575 (93.04)	0.873	0.679–1.122	0.287
*MTHFR* c.1298A>C					
AA	127 (53.59)	381 (61.65)	1		
AC	89 (37.55)	207 (33.50)	1.290	0.937–1.775	0.118
CC	21 (8.86)	30 (4.85)	0.700	0.381–1.288	0.250
AC+CC	110 (46.41)	237 (38.35)	1.392	1.029–1.884	***0.032***
AA+AC	216 (91.14)	588 (95.15)	1.102	0.855–1.421	0.454
*MTRR* c.66A>G					
AA	102 (43.04)	340 (55.02)	1		
AG	112 (47.26)	227 (36.73)	1.645	1.198–2.257	***0.002***
GG	23 (9.70)	51 (8.25)	1.503	0.876–2.579	0.137
AG+GG	135 (56.96)	278 (44.98)	1.619	1.197–2.190	***0.002***
AA+AG	214 (90.30)	567 (91.75)	1.258	0.959–1.650	0.097

^a^ Logistic regression analysis and the chi-square test were used to evaluate the data with 95% CIs.

Significant findings are shown in bold-italic font. The dominant model refers to that heterozygous or homozygous variants at a specific locus will exhibit corresponding phenotypes, for example, AG and GG genotypes of c.66 in *MTRR* are both RPL risk factors.

## Discussion

RPL is a frequent obstetric complication that includes coagulation disorders, autoimmune defects, endocrine disorders, and endometrial defects. An estimated 1–2% of women will present with three or more miscarriages during their reproductive years, and almost 5% of women will present with two or more miscarriages (Larsen *et al.*, 2013; Abu-Heija, 2014). Although RPL has been well studied, the exact etiology remains largely unknown. Several recent studies have concentrated on the association between genetic variants of folic acid-methionine metabolic circulation and RPL.

In the present study, we found three variants in folic acid-methionine metabolic circulation associated with RPL susceptibility, in which T allele of *MTHFR* c.677 was a protective allele and C allele of *MTHFR* c.1298 and G allele of *MTRR* c.66 was a risk allele in RPL in the Chinese population. To our knowledge, our study is the first to investigate the association between c.66A>G in *MTRR* and RPL susceptibility in a Chinese Han population residing in South China. As studies have illustrated, MTRR is the reductase that maintains the activation state of MTR, which plays an important role in the folate cycle. Moreover, MTRR is also critical for the utilization of methyl groups, which requires the methylation of DNA and histones from the folate cycle (Kasak *et al.*, 2017). Therefore, once this vital enzyme was disrupted in folate metabolism, the epigenetic regulation of many genes and entire pathways may be significantly affected. Experiments in mice suggested that MTRR deficiency had a transgenerational effect by epigenetic markers and could cause growth defects and congenital malformations (Padmanabhan *et al.*, 2013). In MTRR, an isoleucine-to-methionine change when c.66A>G occurred, which is its most common variant (Yousefian *et al.*, 2014). Even though a few studies have studied the relationship between the variant c.66A>G in *MTRR* and RPL, their results are still controversial. Consistent with Zhu *et al.*'s study, the G allele of *MTRR* c.66A>G was the risk factor of RPL in our present study (Zhu *et al.*, 2015). This suggested that the distributions of genotype and allele frequencies may be different among different districts.

The role of MTHFR in the conversion of 5,10-methylenetetrahydrofolate to 5-methylenetetrahydrofolate and regulation of the methylation of HCY methionine is clear. However, the exact effect of *MTHFR* c.677C>T to MTHFR was still unclear and controversial. Frosst *et al.* found TT homozygote showed ∼30% enzyme activity, which was significantly lower than that of CT heterozygote (almost 65% of normal enzyme activity) and CC homozygote (normal enzyme activity), which suggested *MTHFR* c.677C>T could affect enzyme activity (Frosst *et al.*, 1995). Moreover, some studies have shown that *MTHFR* gene c.677C>T allele is associated with mild hyperhomocysteinemia and RPL (Nelen *et al.*, 1997; Jeddi-Tehrani *et al.*, 2011). Contrary to these studies, which showed that the c.677C>T allele was associated with RPL disease, in our study, the frequency of the c.677 T allele was significantly lower in women suffering from RPL than that in healthy controls, which indicated that the T allele was associated with lower susceptibility to RPL and could be a protective factor for RPL. Our study was consistent with Makino's study, but in contrast to Mtiraoui's work (Makino *et al.*, 2004; Mtiraoui *et al.*, 2006; Abu-Heija, 2014). In the study by Mtiraoui *et al.*, the T allele was higher in women who lost their fetuses than in those with uneventful pregnancies. Moreover, no significant association between this variant and RPL was reported in some studies (Kobashi *et al.*, 2005; Bae *et al.*, 2009). These divergent findings may be due to the differences in the ethnicity of the women studied and variations in gestational age at RPL. The other mutant allele, c.1298A>C, was also in the *MTHFR* gene, which has an effect on enzyme regulation, probably via *S*-adenosylmethionine, an allosteric inhibitor of MTHFR (Rozen, 1996). Similar to variant c.677C>T, the results of reported studies were controversial. In our study, the frequency of the c.1298 C allele was significantly higher in RPL women than in the control group, and the C allele presented a risk factor for RPL susceptibility. This result was supported by the work of Abu-Heija (2014) and Zetterberg *et al.* (2002).

According to gnomAD 2.0 database, allele frequency of *MTHFR* c.677 C > T in East Asian population is 0.29, while in South Asia 0.15, Europe 0.33, and Ashkenazi Jewish 0.45, respectively; allele frequency of *MTHFR* c. 1298 A > C in East Asian population is 0.22, while the frequencies of the South Asian, European, and Ashkenazi Jewish populations are all higher than 0.29. The distinct allele frequency in separated population indicated that the two genotypes of c.677 C > T and c.1298 A>C loci are not linked but act as two independent genetic events. Allele frequency of *MTRR* c.66 A>G in East Asian is 0.28, whereas South Asian, European, and Ashkenazi Jewish populations have frequencies above 0.52. Differences in genotype frequencies among populations may also be partly responsible for the discrepancy in research findings.

In summary, our study found that variant c.677C>T of *MTHFR* was associated with decreased RPL susceptibility, but variant c.1298A>C of *MTHFR* and the c.66A>G of *MTRR* were associated with increased RPL susceptibility in a population in southern China. *MTHFR* c.1298 C and *MTRR* c.66 G could be designated as minor alleles, while *MTHFR* c.677C>T may be more prevalent in women with uneventful pregnancies and could be considered an ancestral allele. It is worthwhile to mention that there are several limitations in this present study. The sample size is relatively small, and only Han Chinese women in southern China were recruited. Therefore, further larger multicenter case–control studies and other ethnic populations are required to fully explore the role of these factors in causing RPL. However, even with this limitation, the major clinical implication of our findings is that appropriately targeted folic acid supplements may be beneficial for preventing RPL. Targeted folate supplements could reduce thrombosis-related pregnancy loss by catalyzing HCY remethylation into methionine and thereby reduce plasma HCY levels.

## Supplementary Material

Supplemental data
