# Supplementary material for: Variants c.677 C>T, c.1298 A>C in MTHFR, and c.66 A>G in MTRR Affect the Occurrence of Recurrent Pregnancy Loss in Chinese Women
Source: Genet Test Mol Biomarkers. 2020 Nov 10;24(11):717–22. doi: 10.1089/gtmb.2020.0106 (PMC7698989; doi:10.1089/gtmb.2020.0106)
Supplement: Supplemental data [file Supp_Table1.pdf]

Supplementary Table S1. Information for the Subjects

patient No.	age	C677T(MTHFR)	A1298C(MTHFR)	A66G(MTRR)	group	abortion times	uric acid	HCY	total cholesterol
patient 1	25	1	2	2	0	0	#N/A	#N/A	#N/A
patient 2	28	1	1	3	0	0	#N/A	#N/A	#N/A
patient 3	33	2	1	1	0	0	#N/A	#N/A	#N/A
patient 4	29	2	1	1	0	0	#N/A	#N/A	#N/A
patient 5	31	1	1	1	0	0	#N/A	#N/A	#N/A
patient 6	27	1	1	3	0	0	#N/A	#N/A	#N/A
patient 7	26	2	1	1	0	0	#N/A	#N/A	#N/A
patient 8	26	1	1	1	0	0	#N/A	#N/A	#N/A
patient 9	29	1	2	1	0	0	#N/A	#N/A	#N/A
patient 10	26	1	2	2	0	0	#N/A	#N/A	#N/A
patient 11	28	1	1	3	1	3	#N/A	#N/A	#N/A
patient 12	29	1	1	1	0	0	#N/A	#N/A	#N/A
patient 13	32	1	2	1	0	0	#N/A	#N/A	#N/A
patient 14	31	2	1	1	0	0	#N/A	#N/A	#N/A
patient 15	27	1	2	2	1	3	#N/A	#N/A	#N/A
patient 16	28	1	1	1	1	2	#N/A	#N/A	#N/A
patient 17	27	1	2	1	1	2	#N/A	#N/A	#N/A
patient 18	23	1	2	1	1	2	#N/A	#N/A	#N/A
patient 19	29	2	2	3	0	0	#N/A	#N/A	#N/A
patient 20	32	1	3	1	0	0	#N/A	#N/A	#N/A
patient 21	33	2	1	1	0	0	#N/A	#N/A	#N/A
patient 22	25	1	2	2	0	0	#N/A	#N/A	#N/A
patient 23	30	1	2	1	1	2	#N/A	#N/A	#N/A
patient 24	26	1	2	1	0	0	#N/A	#N/A	#N/A
patient 25	26	2	1	2	1	2	#N/A	#N/A	#N/A
patient 26	26	1	1	2	0	0	#N/A	#N/A	#N/A
patient 27	39	2	2	1	0	0	#N/A	#N/A	#N/A
patient 28	25	1	2	1	1	2	#N/A	#N/A	#N/A
patient 29	24	2	1	1	0	0	#N/A	#N/A	#N/A
patient 30	37	1	3	1	1	2	223.2	#N/A	#N/A
patient 31	27	2	1	1	0	0	#N/A	#N/A	#N/A
patient 32	32	2	1	1	0	0	#N/A	#N/A	#N/A
patient 33	25	1	2	2	1	2	#N/A	#N/A	#N/A
patient 34	28	2	1	1	0	0	#N/A	#N/A	#N/A
patient 35	28	1	1	1	0	0	207.1	#N/A	#N/A
patient 36	26	1	2	2	1	2	#N/A	#N/A	#N/A
patient 37	42	2	1	1	0	0	#N/A	#N/A	#N/A
patient 38	20	1	3	2	0	0	197.4	#N/A	6.67
patient 39	33	1	1	2	1	2	#N/A	#N/A	#N/A
patient 40	29	1	2	3	0	0	220.05	#N/A	4.56
patient 41	27	1	1	1	1	2	143.8	#N/A	#N/A
patient 42	34	1	1	1	1	2	225.6	#N/A	5.57
patient 43	28	2	1	2	0	0	#N/A	#N/A	#N/A
patient 44	21	1	1	2	0	0	207.5	#N/A	#N/A
patient 45	25	1	2	2	1	3	310.8	#N/A	5.23
patient 46	24	1	2	1	0	0	#N/A	#N/A	#N/A
patient 47	30	2	1	2	1	2	#N/A	#N/A	#N/A
patient 48	23	2	1	2	1	2	#N/A	#N/A	#N/A
patient 49	23	2	2	1	1	2	#N/A	#N/A	#N/A
patient 50	28	3	1	1	0	0	132.25	#N/A	4.58
patient 51	23	1	1	1	1	2	174.4	#N/A	6.58
patient 52	26	1	3	1	0	0	#N/A	#N/A	#N/A
patient 53	23	1	2	3	1	2	#N/A	#N/A	#N/A
patient 54	27	1	1	1	0	0	#N/A	#N/A	#N/A
patient 55	32	1	2	3	0	0	#N/A	#N/A	#N/A
patient 56	32	1	3	2	0	0	#N/A	#N/A	#N/A
patient 57	28	1	1	2	0	0	#N/A	#N/A	#N/A
patient 58	24	2	1	1	0	0	#N/A	#N/A	#N/A
patient 59	34	2	1	2	0	0	#N/A	#N/A	#N/A
patient 60	31	1	1	1	0	0	185.55	#N/A	5.11
patient 61	27	1	2	2	0	0	202.4	#N/A	4.3
patient 62	27	2	1	2	0	0	#N/A	#N/A	#N/A
patient 63	30	3	1	1	1	2	150.8	#N/A	5.48
patient 64	23	1	2	1	1	3	#N/A	#N/A	#N/A
patient 65	26	1	2	1	0	0	#N/A	#N/A	#N/A
patient 66	26	2	1	1	0	0	#N/A	#N/A	#N/A
patient 67	21	1	1	2	0	0	#N/A	#N/A	#N/A
patient 68	22	1	2	1	0	0	258.6	#N/A	5.93
patient 69	30	2	2	2	0	0	178.6	#N/A	5.26
patient 70	27	2	2	1	0	0	#N/A	#N/A	#N/A
patient 71	22	1	1	1	0	0	#N/A	#N/A	#N/A
patient 72	26	1	1	3	0	0	#N/A	#N/A	#N/A
patient 73	25	1	1	1	0	0	#N/A	#N/A	#N/A
patient 74	29	2	2	2	0	0	#N/A	#N/A	#N/A
patient 75	29	1	2	2	0	0	146.7	#N/A	3.87
patient 76	25	2	2	1	0	0	#N/A	#N/A	#N/A
patient 77	24	2	1	2	0	0	#N/A	#N/A	#N/A
patient 78	24	2	2	1	0	0	#N/A	#N/A	#N/A
patient 79	30	3	1	1	1	2	#N/A	#N/A	#N/A
patient 80	27	3	1	2	0	0	142	#N/A	3.39
patient 81	26	2	1	1	0	0	229.55	#N/A	5.24
patient 82	27	1	2	3	0	0	166.8	#N/A	#N/A
patient 83	28	3	1	2	0	0	#N/A	#N/A	#N/A
patient 84	24	2	2	2	0	0	119.3	#N/A	6.41
patient 85	29	1	1	1	0	0	#N/A	#N/A	#N/A
patient 86	35	1	1	1	0	0	#N/A	#N/A	#N/A
patient 87	26	1	2	2	0	0	184	#N/A	5.63
patient 88	25	1	1	2	0	0	143.75	#N/A	5.74
patient 89	27	2	1	2	0	0	#N/A	#N/A	#N/A
patient 90	37	2	1	1	1	2	#N/A	#N/A	#N/A
patient 91	20	1	3	1	0	0	#N/A	#N/A	#N/A
patient 92	22	2	1	2	0	0	204.3	#N/A	7.67
patient 93	33	2	2	1	0	0	114.4	#N/A	4.22

group: 0 for control， 1 for abortion

patient 94	17	2	1	1	0	0	#N/A	#N/A	#N/A
patient 95	34	1	1	3	0	0	#N/A	#N/A	#N/A
patient 96	29	1	1	1	0	0	#N/A	#N/A	#N/A
patient 97	31	2	1	2	0	0	#N/A	#N/A	#N/A
patient 98	30	3	1	1	0	0	#N/A	#N/A	#N/A
patient 99	34	3	1	1	0	0	#N/A	#N/A	#N/A
patient 100	28	1	2	2	0	0	109.7	#N/A	#N/A
patient 101	32	1	2	2	1	2	#N/A	#N/A	#N/A
patient 102	24	1	1	2	1	3	183.4	#N/A	#N/A
patient 103	37	1	1	2	1	3	83.2	#N/A	#N/A
patient 104	27	1	3	1	0	0	#N/A	#N/A	#N/A
patient 105	18	2	1	1	0	0	#N/A	#N/A	#N/A
patient 106	31	1	1	1	1	2	#N/A	#N/A	#N/A
patient 107	28	2	2	2	1	2	#N/A	#N/A	#N/A
patient 108	33	1	2	1	0	0	#N/A	#N/A	#N/A
patient 109	28	1	3	1	1	2	242.2	#N/A	#N/A
patient 110	27	1	1	2	1	2	#N/A	#N/A	#N/A
patient 111	32	2	1	1	0	0	#N/A	#N/A	#N/A
patient 112	28	2	2	2	0	0	#N/A	#N/A	#N/A
patient 113	20	2	1	2	0	0	#N/A	#N/A	#N/A
patient 114	27	2	1	2	1	3	#N/A	#N/A	#N/A
patient 115	25	1	1	2	0	0	#N/A	#N/A	#N/A
patient 116	27	1	1	1	0	0	#N/A	#N/A	#N/A
patient 117	25	2	2	1	0	0	#N/A	#N/A	#N/A
patient 118	31	2	1	2	0	0	#N/A	#N/A	#N/A
patient 119	21	2	2	1	1	3	#N/A	#N/A	#N/A
patient 120	29	1	3	1	0	0	#N/A	#N/A	#N/A
patient 121	22	2	1	2	0	0	#N/A	#N/A	#N/A
patient 122	19	1	3	2	1	2	334	#N/A	4.26
patient 123	25	1	2	2	1	2	#N/A	#N/A	#N/A
patient 124	24	2	2	2	0	0	236.5	#N/A	5.65
patient 125	31	1	2	2	1	2	249.33	4.2	5.84
patient 126	27	2	1	2	0	0	291.75	#N/A	9.15
patient 127	30	2	1	2	0	0	234.4	#N/A	#N/A
patient 128	24	1	1	1	0	0	207.7	#N/A	3.98
patient 129	25	1	2	2	0	0	#N/A	#N/A	#N/A
patient 130	24	2	2	2	0	0	#N/A	#N/A	#N/A
patient 131	18	2	1	1	1	2	#N/A	#N/A	#N/A
patient 132	31	2	2	2	1	2	#N/A	#N/A	#N/A
patient 133	29	1	1	2	0	0	#N/A	#N/A	#N/A
patient 134	27	1	1	1	0	0	#N/A	#N/A	#N/A
patient 135	30	1	1	1	1	2	#N/A	#N/A	#N/A
patient 136	30	1	1	2	0	0	#N/A	#N/A	#N/A
patient 137	28	1	2	3	0	0	#N/A	#N/A	3.54
patient 138	29	1	2	2	1	2	#N/A	#N/A	#N/A
patient 139	26	2	1	3	0	0	#N/A	#N/A	#N/A
patient 140	31	2	2	1	0	0	#N/A	#N/A	#N/A
patient 141	36	1	1	2	0	0	#N/A	#N/A	#N/A
patient 142	28	1	1	2	1	2	#N/A	#N/A	#N/A
patient 143	21	1	1	2	0	0	#N/A	#N/A	#N/A
patient 144	28	1	1	1	0	0	#N/A	#N/A	#N/A
patient 145	28	1	1	2	0	0	#N/A	#N/A	#N/A
patient 146	37	1	1	2	0	0	#N/A	#N/A	#N/A
patient 147	37	2	2	2	0	0	#N/A	#N/A	#N/A
patient 148	28	2	1	1	1	2	#N/A	#N/A	#N/A
patient 149	24	1	2	2	1	2	#N/A	#N/A	#N/A
patient 150	33	2	1	2	1	2	#N/A	#N/A	#N/A
patient 151	29	1	1	2	1	2	#N/A	#N/A	#N/A
patient 152	24	1	2	1	0	0	238.8	#N/A	5.31
patient 153	30	2	2	2	1	3	#N/A	#N/A	#N/A
patient 154	30	2	1	2	0	0	174	#N/A	4.7
patient 155	30	1	2	1	1	2	#N/A	#N/A	#N/A
patient 156	22	2	2	1	0	0	#N/A	#N/A	#N/A
patient 157	23	2	1	1	0	0	#N/A	#N/A	#N/A
patient 158	25	2	1	2	1	2	#N/A	#N/A	#N/A
patient 159	26	2	2	1	1	2	66	#N/A	#N/A
patient 160	23	3	1	1	0	0	#N/A	#N/A	#N/A
patient 161	34	1	1	1	1	2	#N/A	#N/A	#N/A
patient 162	33	1	2	1	0	0	#N/A	#N/A	#N/A
patient 163	33	3	1	2	1	2	245.4	#N/A	5.51
patient 164	25	1	2	2	0	0	247	#N/A	5.71
patient 165	29	2	1	2	1	2	#N/A	#N/A	#N/A
patient 166	29	2	1	1	0	0	#N/A	#N/A	#N/A
patient 167	25	1	2	1	0	0	176.2	#N/A	#N/A
patient 168	28	1	2	2	0	0	#N/A	#N/A	#N/A
patient 169	30	1	2	2	1	2	#N/A	#N/A	#N/A
patient 170	22	2	2	1	1	3	#N/A	#N/A	#N/A
patient 171	30	1	2	1	1	2	122.55	#N/A	4.06
patient 172	22	1	2	1	0	0	#N/A	#N/A	#N/A
patient 173	27	2	1	1	0	0	#N/A	#N/A	#N/A
patient 174	29	1	2	1	0	0	#N/A	#N/A	#N/A
patient 175	25	1	1	1	0	0	#N/A	#N/A	#N/A
patient 176	23	2	1	1	0	0	#N/A	#N/A	#N/A
patient 177	26	1	1	2	0	0	#N/A	#N/A	#N/A
patient 178	30	2	2	3	0	0	163	#N/A	#N/A
patient 179	30	1	1	1	1	2	#N/A	#N/A	#N/A
patient 180	33	3	1	1	0	0	#N/A	#N/A	#N/A
patient 181	29	1	2	2	0	0	191.53	6.5	6.32
patient 182	33	2	2	1	0	0	#N/A	#N/A	#N/A
patient 183	27	2	1	3	1	2	242.2	#N/A	#N/A
patient 184	22	1	3	2	0	0	168.2	5.3	5.22
patient 185	25	1	2	2	1	2	#N/A	#N/A	#N/A
patient 186	26	1	2	2	1	2	#N/A	#N/A	#N/A
patient 187	27	1	1	2	1	2	#N/A	#N/A	#N/A
patient 188	22	2	1	1	0	0	#N/A	#N/A	#N/A

patient 189	29	2	1	2	0	0	#N/A	#N/A	5.89
patient 190	24	2	1	1	0	0	#N/A	#N/A	#N/A
patient 191	36	1	2	1	1	2	#N/A	#N/A	#N/A
patient 192	28	1	2	1	1	2	#N/A	#N/A	#N/A
patient 193	31	1	2	1	0	0	208.55	5.6	5.48
patient 194	28	1	3	1	1	2	#N/A	#N/A	#N/A
patient 195	28	1	2	3	1	2	#N/A	#N/A	#N/A
patient 196	32	1	2	2	1	3	#N/A	#N/A	#N/A
patient 197	34	1	3	1	1	2	#N/A	#N/A	#N/A
patient 198	29	1	2	1	0	0	#N/A	#N/A	#N/A
patient 199	31	2	1	2	0	0	196	#N/A	#N/A
patient 200	25	1	1	1	0	0	#N/A	#N/A	#N/A
patient 201	34	2	2	1	0	0	#N/A	#N/A	#N/A
patient 202	25	1	2	2	0	0	#N/A	#N/A	#N/A
patient 203	24	1	1	2	0	0	231.1	#N/A	4.26
patient 204	31	2	1	1	0	0	170.1	#N/A	#N/A
patient 205	24	3	1	2	1	2	#N/A	#N/A	#N/A
patient 206	31	2	1	3	1	3	#N/A	#N/A	#N/A
patient 207	27	2	1	2	0	0	216.3	#N/A	#N/A
patient 208	21	1	2	2	1	2	#N/A	#N/A	#N/A
patient 209	23	2	1	2	1	3	#N/A	#N/A	#N/A
patient 210	31	1	1	1	0	0	#N/A	#N/A	#N/A
patient 211	24	1	1	2	1	2	#N/A	#N/A	#N/A
patient 212	25	1	1	2	0	0	#N/A	#N/A	#N/A
patient 213	23	1	1	1	1	2	#N/A	#N/A	#N/A
patient 214	32	2	1	3	1	2	#N/A	#N/A	#N/A
patient 215	28	1	3	1	1	2	#N/A	#N/A	#N/A
patient 216	30	2	1	2	0	0	#N/A	#N/A	#N/A
patient 217	26	2	2	2	0	0	#N/A	#N/A	#N/A
patient 218	28	1	2	1	0	0	#N/A	#N/A	#N/A
patient 219	26	2	1	2	0	0	#N/A	#N/A	#N/A
patient 220	24	1	2	1	1	3	#N/A	#N/A	#N/A
patient 221	28	1	1	1	1	3	#N/A	#N/A	#N/A
patient 222	22	1	2	1	0	0	#N/A	#N/A	#N/A
patient 223	28	1	1	2	0	0	#N/A	#N/A	#N/A
patient 224	24	1	1	1	0	0	#N/A	#N/A	#N/A
patient 225	30	3	1	1	0	0	123.9	#N/A	4.65
patient 226	24	1	2	1	1	2	#N/A	#N/A	#N/A
patient 227	30	2	1	2	0	0	#N/A	#N/A	#N/A
patient 228	30	2	1	1	1	2	#N/A	#N/A	#N/A
patient 229	35	2	1	1	1	2	#N/A	#N/A	#N/A
patient 230	25	2	1	2	1	2	#N/A	#N/A	#N/A
patient 231	25	1	1	3	0	0	#N/A	#N/A	#N/A
patient 232	23	2	1	1	0	0	195.1	#N/A	#N/A
patient 233	28	1	1	2	1	2	#N/A	#N/A	#N/A
patient 234	25	2	1	2	1	2	#N/A	#N/A	#N/A
patient 235	35	2	1	2	0	0	#N/A	#N/A	#N/A
patient 236	24	2	2	2	0	0	#N/A	#N/A	#N/A
patient 237	26	2	2	2	0	0	218.3	#N/A	4.69
patient 238	25	2	2	3	0	0	#N/A	#N/A	#N/A
patient 239	37	1	1	3	0	0	#N/A	#N/A	#N/A
patient 240	24	1	2	1	0	0	#N/A	#N/A	#N/A
patient 241	22	2	1	2	0	0	#N/A	#N/A	#N/A
patient 242	23	2	2	1	1	2	#N/A	#N/A	#N/A
patient 243	29	2	2	1	0	0	#N/A	#N/A	#N/A
patient 244	23	2	1	1	0	0	#N/A	#N/A	#N/A
patient 245	30	1	1	2	1	4	#N/A	#N/A	#N/A
patient 246	23	2	2	1	1	2	#N/A	#N/A	#N/A
patient 247	22	1	2	2	0	0	271.5	#N/A	6.29
patient 248	26	2	2	1	0	0	192.5	#N/A	4.56
patient 249	38	1	2	2	1	2	#N/A	#N/A	#N/A
patient 250	27	1	3	2	0	0	#N/A	#N/A	#N/A
patient 251	26	2	1	1	0	0	#N/A	#N/A	#N/A
patient 252	22	1	1	1	0	0	#N/A	#N/A	#N/A
patient 253	26	1	2	2	0	0	#N/A	#N/A	#N/A
patient 254	38	1	2	1	1	2	229.4	#N/A	5.05
patient 255	26	1	2	1	0	0	#N/A	#N/A	#N/A
patient 256	24	2	1	2	0	0	#N/A	#N/A	#N/A
patient 257	25	1	2	1	1	3	#N/A	#N/A	#N/A
patient 258	40	2	2	3	1	2	#N/A	#N/A	#N/A
patient 259	32	2	1	1	0	0	#N/A	#N/A	#N/A
patient 260	26	1	3	2	0	0	#N/A	#N/A	#N/A
patient 261	30	1	2	2	0	0	#N/A	#N/A	#N/A
patient 262	27	1	2	2	1	3	#N/A	#N/A	#N/A
patient 263	31	1	1	2	0	0	#N/A	#N/A	#N/A
patient 264	29	1	2	3	0	0	#N/A	#N/A	#N/A
patient 265	26	1	1	1	0	0	#N/A	#N/A	#N/A
patient 266	28	2	1	3	1	2	#N/A	#N/A	#N/A
patient 267	40	2	2	2	1	2	#N/A	#N/A	#N/A
patient 268	32	1	1	1	0	0	505.8	#N/A	#N/A
patient 269	42	1	1	1	1	2	#N/A	#N/A	#N/A
patient 270	31	1	1	1	0	0	101.7	#N/A	5.16
patient 271	24	1	2	1	0	0	#N/A	#N/A	#N/A
patient 272	27	3	1	2	0	0	#N/A	#N/A	#N/A
patient 273	26	2	1	2	0	0	#N/A	#N/A	#N/A
patient 274	28	2	1	1	0	0	274.6	#N/A	7.8
patient 275	22	1	2	1	1	2	148.6	#N/A	#N/A
patient 276	27	1	2	2	1	3	#N/A	#N/A	#N/A
patient 277	31	2	2	1	0	0	209.7	#N/A	#N/A
patient 278	31	1	2	1	0	0	#N/A	#N/A	#N/A
patient 279	26	1	1	3	0	0	#N/A	#N/A	#N/A
patient 280	22	1	2	1	0	0	#N/A	#N/A	#N/A
patient 281	32	1	1	1	0	0	#N/A	#N/A	#N/A
patient 282	24	2	1	1	0	0	#N/A	#N/A	#N/A
patient 283	21	1	2	1	1	2	#N/A	#N/A	#N/A

patient 284	24	3	1	1	0	0	#N/A	#N/A	#N/A
patient 285	27	1	1	2	1	2	#N/A	#N/A	#N/A
patient 286	21	1	2	2	0	0	#N/A	#N/A	#N/A
patient 287	26	2	1	3	0	0	254.9	#N/A	#N/A
patient 288	35	1	1	1	0	0	#N/A	#N/A	#N/A
patient 289	29	2	1	3	0	0	#N/A	#N/A	#N/A
patient 290	27	1	1	2	0	0	#N/A	#N/A	#N/A
patient 291	31	1	1	1	0	0	#N/A	#N/A	#N/A
patient 292	26	3	1	2	0	0	#N/A	#N/A	#N/A
patient 293	27	2	1	2	0	0	201.9	#N/A	6.1
patient 294	24	2	1	3	1	2	#N/A	#N/A	#N/A
patient 295	23	2	1	1	0	0	#N/A	#N/A	#N/A
patient 296	26	2	1	1	0	0	225.1	7.1	#N/A
patient 297	25	2	2	3	0	0	#N/A	#N/A	#N/A
patient 298	27	2	1	2	0	0	#N/A	#N/A	#N/A
patient 299	32	1	1	2	0	0	#N/A	#N/A	#N/A
patient 300	26	2	2	1	1	2	#N/A	#N/A	#N/A
patient 301	25	2	1	2	0	0	99.8	#N/A	3.95
patient 302	32	2	1	3	1	2	#N/A	#N/A	#N/A
patient 303	26	1	1	2	0	0	#N/A	#N/A	#N/A
patient 304	30	1	3	1	0	0	217.8	#N/A	#N/A
patient 305	31	3	1	2	0	0	#N/A	#N/A	#N/A
patient 306	26	2	2	1	1	2	#N/A	#N/A	#N/A
patient 307	26	1	2	1	0	0	#N/A	#N/A	#N/A
patient 308	22	3	1	1	0	0	#N/A	#N/A	#N/A
patient 309	23	1	1	3	0	0	#N/A	#N/A	#N/A
patient 310	22	2	1	1	0	0	347.7	#N/A	5.93
patient 311	36	1	2	1	0	0	#N/A	#N/A	#N/A
patient 312	23	1	1	2	1	2	176.8	#N/A	6.37
patient 313	29	1	2	1	0	0	#N/A	#N/A	#N/A
patient 314	33	1	2	2	1	2	#N/A	#N/A	#N/A
patient 315	23	2	1	2	1	2	#N/A	#N/A	#N/A
patient 316	30	1	1	1	1	3	#N/A	#N/A	#N/A
patient 317	23	2	2	3	0	0	115.1	#N/A	4.19
patient 318	32	2	1	1	0	0	227.1	#N/A	#N/A
patient 319	28	1	3	1	1	2	#N/A	#N/A	#N/A
patient 320	22	2	2	1	0	0	#N/A	#N/A	#N/A
patient 321	28	2	1	2	0	0	307.95	#N/A	5.56
patient 322	32	2	1	2	1	2	#N/A	6.5	#N/A
patient 323	25	1	2	2	0	0	#N/A	#N/A	#N/A
patient 324	30	2	1	3	0	0	#N/A	#N/A	#N/A
patient 325	31	2	1	1	0	0	119.2	#N/A	4.05
patient 326	25	2	1	2	1	2	#N/A	#N/A	#N/A
patient 327	20	2	1	1	0	0	152.2	#N/A	4.54
patient 328	34	3	1	2	0	0	#N/A	#N/A	#N/A
patient 329	28	2	1	3	0	0	#N/A	#N/A	#N/A
patient 330	27	1	2	1	0	0	#N/A	#N/A	#N/A
patient 331	25	2	1	1	0	0	#N/A	#N/A	#N/A
patient 332	29	1	3	2	0	0	#N/A	#N/A	#N/A
patient 333	20	2	1	1	0	0	#N/A	#N/A	#N/A
patient 334	22	2	1	1	0	0	#N/A	#N/A	#N/A
patient 335	30	1	1	1	1	3	#N/A	#N/A	#N/A
patient 336	26	1	1	1	0	0	#N/A	#N/A	#N/A
patient 337	27	1	2	1	0	0	165.9	#N/A	4.81
patient 338	24	2	2	1	0	0	#N/A	8.5	#N/A
patient 339	20	1	1	1	0	0	#N/A	#N/A	#N/A
patient 340	28	2	2	1	0	0	#N/A	#N/A	#N/A
patient 341	26	2	1	1	1	2	#N/A	#N/A	#N/A
patient 342	32	2	1	1	1	2	#N/A	#N/A	#N/A
patient 343	24	1	1	2	0	0	#N/A	#N/A	#N/A
patient 344	25	2	1	1	0	0	#N/A	#N/A	#N/A
patient 345	31	1	1	2	0	0	#N/A	#N/A	#N/A
patient 346	24	1	2	2	0	0	#N/A	#N/A	#N/A
patient 347	22	1	2	1	0	0	#N/A	#N/A	#N/A
patient 348	24	1	1	1	0	0	#N/A	#N/A	#N/A
patient 349	29	1	1	2	1	2	#N/A	#N/A	#N/A
patient 350	32	1	1	1	0	0	#N/A	#N/A	#N/A
patient 351	20	1	1	2	0	0	#N/A	#N/A	#N/A
patient 352	29	2	2	2	0	0	144.5	#N/A	#N/A
patient 353	28	1	2	1	0	0	#N/A	#N/A	#N/A
patient 354	25	2	2	2	0	0	#N/A	#N/A	#N/A
patient 355	23	1	1	2	0	0	#N/A	#N/A	#N/A
patient 356	21	2	1	2	0	0	#N/A	#N/A	#N/A
patient 357	26	1	1	2	1	3	#N/A	#N/A	#N/A
patient 358	23	1	3	1	0	0	#N/A	#N/A	#N/A
patient 359	31	3	1	2	0	0	#N/A	#N/A	#N/A
patient 360	25	1	1	1	0	0	#N/A	#N/A	#N/A
patient 361	28	1	1	1	0	0	#N/A	#N/A	#N/A
patient 362	26	1	2	1	0	0	156.7	#N/A	4.33
patient 363	25	2	1	1	0	0	#N/A	#N/A	#N/A
patient 364	27	2	1	2	0	0	#N/A	#N/A	#N/A
patient 365	23	1	1	3	1	3	#N/A	#N/A	#N/A
patient 366	30	3	1	1	0	0	#N/A	#N/A	#N/A
patient 367	29	1	1	3	0	0	#N/A	#N/A	#N/A
patient 368	30	2	1	2	0	0	#N/A	#N/A	#N/A
patient 369	23	1	1	2	0	0	#N/A	#N/A	#N/A
patient 370	25	1	2	3	0	0	164.3	#N/A	5.42
patient 371	36	1	1	2	0	0	320.1	#N/A	5.27
patient 372	28	1	1	2	0	0	#N/A	#N/A	#N/A
patient 373	33	2	1	3	0	0	#N/A	#N/A	#N/A
patient 374	26	2	1	1	0	0	161.5	#N/A	3.66
patient 375	23	2	2	2	0	0	#N/A	#N/A	#N/A
patient 376	31	1	2	1	0	0	#N/A	#N/A	#N/A
patient 377	26	2	1	1	0	0	#N/A	#N/A	#N/A
patient 378	31	2	1	1	0	0	#N/A	#N/A	#N/A

patient 379	22	2	1	2	0	0	#N/A	#N/A	#N/A
patient 380	29	2	1	1	0	0	217	#N/A	#N/A
patient 381	37	1	1	2	0	0	#N/A	#N/A	#N/A
patient 382	23	1	2	2	0	0	#N/A	#N/A	#N/A
patient 383	28	1	2	3	0	0	#N/A	#N/A	#N/A
patient 384	25	1	2	2	0	0	#N/A	#N/A	#N/A
patient 385	24	1	2	2	0	0	#N/A	#N/A	#N/A
patient 386	21	3	1	1	0	0	#N/A	#N/A	#N/A
patient 387	28	3	1	1	0	0	#N/A	#N/A	#N/A
patient 388	22	2	1	3	0	0	#N/A	#N/A	#N/A
patient 389	26	1	2	2	0	0	#N/A	#N/A	#N/A
patient 390	27	2	1	2	0	0	#N/A	#N/A	#N/A
patient 391	32	2	1	3	0	0	#N/A	#N/A	#N/A
patient 392	24	2	1	1	0	0	#N/A	#N/A	#N/A
patient 393	34	1	2	1	0	0	#N/A	#N/A	#N/A
patient 394	34	1	1	1	0	0	117.55	5.7	4.85
patient 395	21	2	1	1	0	0	#N/A	#N/A	#N/A
patient 396	30	1	2	1	0	0	#N/A	#N/A	#N/A
patient 397	24	1	1	2	0	0	#N/A	#N/A	#N/A
patient 398	28	1	2	2	0	0	#N/A	#N/A	#N/A
patient 399	25	2	1	1	0	0	#N/A	#N/A	#N/A
patient 400	27	1	1	1	0	0	225.2	#N/A	4.99
patient 401	24	1	2	2	0	0	#N/A	#N/A	#N/A
patient 402	29	2	1	2	0	0	#N/A	#N/A	#N/A
patient 403	30	1	2	1	0	0	#N/A	#N/A	#N/A
patient 404	25	2	1	2	0	0	139.35	#N/A	#N/A
patient 405	24	2	1	1	0	0	#N/A	#N/A	#N/A
patient 406	24	2	1	1	0	0	#N/A	#N/A	#N/A
patient 407	31	2	1	1	0	0	#N/A	#N/A	#N/A
patient 408	28	2	1	2	0	0	#N/A	#N/A	#N/A
patient 409	29	1	3	2	0	0	#N/A	#N/A	#N/A
patient 410	28	2	2	1	0	0	#N/A	#N/A	#N/A
patient 411	25	2	1	1	0	0	#N/A	#N/A	#N/A
patient 412	32	2	2	1	0	0	#N/A	#N/A	#N/A
patient 413	26	1	1	1	0	0	#N/A	#N/A	#N/A
patient 414	30	3	1	1	0	0	#N/A	#N/A	#N/A
patient 415	26	2	1	1	0	0	#N/A	#N/A	#N/A
patient 416	22	1	2	1	0	0	142.93	6.25	3.25
patient 417	26	1	2	1	0	0	#N/A	#N/A	4.18
patient 418	31	1	1	2	0	0	#N/A	#N/A	#N/A
patient 419	28	1	2	2	0	0	#N/A	#N/A	#N/A
patient 420	24	1	2	3	1	2	#N/A	#N/A	#N/A
patient 421	41	1	2	2	0	0	#N/A	#N/A	#N/A
patient 422	27	2	2	2	1	2	#N/A	#N/A	#N/A
patient 423	31	1	1	1	0	0	#N/A	#N/A	#N/A
patient 424	23	1	1	1	0	0	#N/A	#N/A	#N/A
patient 425	29	2	1	1	1	2	#N/A	#N/A	#N/A
patient 426	29	1	1	1	0	0	#N/A	#N/A	#N/A
patient 427	28	3	1	1	0	0	#N/A	#N/A	#N/A
patient 428	29	1	1	1	0	0	#N/A	#N/A	#N/A
patient 429	26	1	2	1	0	0	#N/A	#N/A	#N/A
patient 430	31	1	2	2	0	0	#N/A	#N/A	#N/A
patient 431	24	1	2	1	0	0	#N/A	#N/A	#N/A
patient 432	28	1	2	1	0	0	#N/A	#N/A	#N/A
patient 433	33	3	1	1	0	0	#N/A	#N/A	#N/A
patient 434	31	1	2	1	0	0	#N/A	#N/A	#N/A
patient 435	26	2	1	1	1	2	138.6	#N/A	#N/A
patient 436	29	1	1	1	0	0	#N/A	#N/A	#N/A
patient 437	22	1	1	1	0	0	#N/A	#N/A	#N/A
patient 438	29	1	2	2	1	2	#N/A	#N/A	#N/A
patient 439	21	2	1	2	0	0	#N/A	#N/A	#N/A
patient 440	38	1	1	1	0	0	#N/A	#N/A	#N/A
patient 441	23	2	1	1	0	0	191.9	#N/A	4.21
patient 442	29	1	1	1	0	0	#N/A	#N/A	#N/A
patient 443	24	2	1	3	1	2	#N/A	#N/A	#N/A
patient 444	24	2	1	2	0	0	#N/A	#N/A	5.91
patient 445	31	1	1	3	1	2	#N/A	#N/A	#N/A
patient 446	23	1	1	2	1	2	#N/A	#N/A	#N/A
patient 447	33	2	1	1	1	2	#N/A	#N/A	#N/A
patient 448	22	1	1	1	0	0	#N/A	#N/A	#N/A
patient 449	26	1	1	1	0	0	108.85	#N/A	3.69
patient 450	30	2	1	1	0	0	#N/A	#N/A	#N/A
patient 451	23	1	2	1	0	0	#N/A	#N/A	#N/A
patient 452	25	2	1	1	0	0	#N/A	#N/A	#N/A
patient 453	22	2	2	3	0	0	#N/A	#N/A	#N/A
patient 454	31	1	1	1	0	0	#N/A	#N/A	#N/A
patient 455	26	2	1	2	0	0	#N/A	#N/A	#N/A
patient 456	25	1	3	1	0	0	#N/A	#N/A	#N/A
patient 457	25	1	1	1	0	0	#N/A	#N/A	#N/A
patient 458	18	2	1	2	0	0	#N/A	#N/A	#N/A
patient 459	22	1	2	1	1	3	#N/A	#N/A	#N/A
patient 460	24	1	1	2	0	0	#N/A	#N/A	#N/A
patient 461	29	1	3	2	1	2	#N/A	#N/A	#N/A
patient 462	31	2	1	2	0	0	196	#N/A	#N/A
patient 463	27	1	1	3	0	0	112	#N/A	#N/A
patient 464	27	1	2	1	1	2	#N/A	#N/A	#N/A
patient 465	32	2	1	2	0	0	197.8	#N/A	4.34
patient 466	19	1	1	2	1	3	#N/A	#N/A	#N/A
patient 467	23	2	1	2	1	2	#N/A	#N/A	#N/A
patient 468	28	2	2	1	1	2	120.65	#N/A	#N/A
patient 469	25	2	1	2	0	0	#N/A	#N/A	#N/A
patient 470	33	2	2	1	0	0	#N/A	#N/A	#N/A
patient 471	22	3	1	2	1	2	#N/A	#N/A	#N/A
patient 472	28	1	2	2	1	2	#N/A	#N/A	#N/A
patient 473	27	2	1	2	1	2	178.4	#N/A	4.85

patient 474	25	1	1	3	1	2	214.4	#N/A	#N/A
patient 475	33	1	3	2	1	3	300.4	#N/A	#N/A
patient 476	29	1	1	2	1	2	#N/A	#N/A	#N/A
patient 477	27	1	1	2	1	2	#N/A	#N/A	#N/A
patient 478	27	1	1	2	0	0	#N/A	#N/A	#N/A
patient 479	19	1	3	1	1	2	#N/A	#N/A	#N/A
patient 480	33	2	2	1	0	0	#N/A	#N/A	#N/A
patient 481	27	2	2	1	1	2	#N/A	#N/A	#N/A
patient 482	27	1	2	3	1	2	#N/A	#N/A	#N/A
patient 483	34	1	2	2	1	2	#N/A	#N/A	#N/A
patient 484	30	2	1	1	0	0	#N/A	#N/A	#N/A
patient 485	29	1	2	2	1	2	#N/A	#N/A	#N/A
patient 486	30	1	1	1	0	0	#N/A	#N/A	#N/A
patient 487	34	1	3	1	0	0	#N/A	#N/A	#N/A
patient 488	27	1	1	2	1	2	#N/A	#N/A	#N/A
patient 489	30	2	1	2	1	2	#N/A	#N/A	#N/A
patient 490	27	1	1	1	0	0	#N/A	#N/A	#N/A
patient 491	24	1	2	1	0	0	#N/A	#N/A	#N/A
patient 492	25	1	2	2	0	0	#N/A	#N/A	#N/A
patient 493	30	3	1	1	0	0	#N/A	#N/A	#N/A
patient 494	22	2	1	1	0	0	125.9	#N/A	#N/A
patient 495	25	1	2	2	0	0	#N/A	#N/A	#N/A
patient 496	23	1	3	1	0	0	#N/A	#N/A	#N/A
patient 497	29	2	1	2	0	0	#N/A	#N/A	#N/A
patient 498	22	2	2	2	0	0	#N/A	#N/A	#N/A
patient 499	29	1	1	1	0	0	148.2	#N/A	#N/A
patient 500	27	2	1	1	0	0	#N/A	#N/A	#N/A
patient 501	23	2	1	1	0	0	#N/A	#N/A	#N/A
patient 502	30	1	2	1	1	2	#N/A	#N/A	#N/A
patient 503	25	1	1	1	0	0	#N/A	#N/A	#N/A
patient 504	21	2	1	2	0	0	#N/A	#N/A	#N/A
patient 505	22	1	1	1	0	0	#N/A	#N/A	#N/A
patient 506	22	2	1	1	0	0	#N/A	#N/A	#N/A
patient 507	27	1	2	1	0	0	#N/A	#N/A	#N/A
patient 508	21	2	1	3	0	0	229.2	#N/A	#N/A
patient 509	24	2	1	2	0	0	#N/A	#N/A	#N/A
patient 510	31	3	1	2	0	0	#N/A	#N/A	#N/A
patient 511	31	1	1	3	0	0	#N/A	#N/A	#N/A
patient 512	24	2	2	2	1	2	#N/A	#N/A	#N/A
patient 513	26	1	2	1	0	0	#N/A	#N/A	#N/A
patient 514	26	2	1	2	1	2	#N/A	#N/A	#N/A
patient 515	30	2	1	1	0	0	#N/A	#N/A	#N/A
patient 516	35	2	1	1	0	0	#N/A	#N/A	#N/A
patient 517	26	2	2	1	1	2	#N/A	#N/A	#N/A
patient 518	17	1	1	2	0	0	#N/A	#N/A	#N/A
patient 519	28	1	1	2	0	0	#N/A	#N/A	#N/A
patient 520	32	1	1	2	1	3	#N/A	#N/A	#N/A
patient 521	31	1	3	2	1	4	#N/A	#N/A	#N/A
patient 522	34	2	2	1	0	0	167.8	#N/A	#N/A
patient 523	25	1	3	2	0	0	#N/A	#N/A	#N/A
patient 524	28	2	2	2	0	0	#N/A	#N/A	#N/A
patient 525	25	1	1	2	0	0	#N/A	#N/A	#N/A
patient 526	26	1	2	1	0	0	#N/A	#N/A	#N/A
patient 527	30	2	1	1	0	0	#N/A	#N/A	#N/A
patient 528	41	3	1	1	1	2	#N/A	#N/A	#N/A
patient 529	26	2	2	1	0	0	#N/A	#N/A	#N/A
patient 530	27	1	1	1	0	0	#N/A	#N/A	#N/A
patient 531	29	1	3	1	0	0	#N/A	#N/A	#N/A
patient 532	22	1	1	2	1	2	#N/A	#N/A	#N/A
patient 533	27	1	3	3	1	2	#N/A	#N/A	#N/A
patient 534	23	1	1	1	0	0	#N/A	#N/A	#N/A
patient 535	23	1	2	1	0	0	#N/A	#N/A	#N/A
patient 536	30	1	2	2	0	0	#N/A	#N/A	#N/A
patient 537	25	1	2	2	0	0	#N/A	#N/A	#N/A
patient 538	23	1	2	1	0	0	245.4	#N/A	4.55
patient 539	41	2	1	1	0	0	#N/A	#N/A	#N/A
patient 540	27	3	1	2	0	0	#N/A	#N/A	#N/A
patient 541	30	2	2	2	1	2	#N/A	#N/A	#N/A
patient 542	32	1	1	2	0	0	#N/A	#N/A	#N/A
patient 543	30	2	1	1	0	0	#N/A	#N/A	#N/A
patient 544	29	1	1	1	0	0	#N/A	#N/A	#N/A
patient 545	27	2	1	2	0	0	#N/A	#N/A	#N/A
patient 546	33	1	1	1	0	0	179.47	5.8	#N/A
patient 547	28	1	1	1	0	0	159.8	#N/A	#N/A
patient 548	25	1	2	2	1	2	#N/A	#N/A	#N/A
patient 549	26	2	2	3	0	0	#N/A	#N/A	#N/A
patient 550	20	1	2	1	0	0	#N/A	#N/A	#N/A
patient 551	25	2	2	1	0	0	265.7	#N/A	6.19
patient 552	28	2	1	1	0	0	256.8	#N/A	4.28
patient 553	29	2	2	1	1	3	#N/A	#N/A	#N/A
patient 554	28	2	1	1	0	0	#N/A	#N/A	#N/A
patient 555	23	1	2	1	0	0	#N/A	#N/A	#N/A
patient 556	29	1	2	2	0	0	#N/A	#N/A	#N/A
patient 557	38	2	2	1	0	0	233.1	#N/A	#N/A
patient 558	26	1	1	3	0	0	#N/A	#N/A	#N/A
patient 559	27	2	2	3	0	0	#N/A	#N/A	#N/A
patient 560	28	1	2	1	0	0	150.8	#N/A	5.48
patient 561	25	1	1	1	0	0	380.77	6.35	6.73
patient 562	25	1	1	1	0	0	#N/A	#N/A	#N/A
patient 563	37	2	1	1	0	0	#N/A	#N/A	#N/A
patient 564	32	2	2	2	1	3	#N/A	#N/A	#N/A
patient 565	25	1	1	1	1	3	#N/A	#N/A	#N/A
patient 566	20	1	1	2	0	0	#N/A	#N/A	#N/A
patient 567	36	2	2	2	0	0	192	#N/A	3.75
patient 568	23	2	1	2	0	0	#N/A	#N/A	#N/A

patient 569	23	1	2	2	0	0	#N/A	#N/A	#N/A
patient 570	26	1	1	1	0	0	216.3	#N/A	#N/A
patient 571	21	2	1	3	0	0	#N/A	#N/A	#N/A
patient 572	31	3	1	1	0	0	284.95	#N/A	5.65
patient 573	35	2	1	2	0	0	#N/A	#N/A	#N/A
patient 574	26	2	1	1	0	0	#N/A	#N/A	#N/A
patient 575	27	3	1	2	0	0	#N/A	#N/A	#N/A
patient 576	35	2	1	1	1	2	#N/A	#N/A	#N/A
patient 577	32	1	3	2	0	0	#N/A	#N/A	#N/A
patient 578	29	2	1	1	0	0	#N/A	#N/A	#N/A
patient 579	29	1	1	1	0	0	#N/A	#N/A	#N/A
patient 580	21	2	2	2	0	0	#N/A	#N/A	#N/A
patient 581	32	2	2	1	0	0	#N/A	#N/A	#N/A
patient 582	23	2	1	1	0	0	#N/A	#N/A	#N/A
patient 583	26	1	3	2	0	0	#N/A	#N/A	#N/A
patient 584	34	1	1	1	0	0	#N/A	#N/A	#N/A
patient 585	24	2	1	2	0	0	#N/A	#N/A	#N/A
patient 586	22	1	2	1	0	0	#N/A	#N/A	#N/A
patient 587	27	2	2	1	0	0	#N/A	#N/A	#N/A
patient 588	22	3	1	2	0	0	#N/A	#N/A	#N/A
patient 589	22	2	2	1	0	0	#N/A	#N/A	#N/A
patient 590	30	2	1	1	0	0	#N/A	#N/A	#N/A
patient 591	30	1	2	2	0	0	132.5	#N/A	#N/A
patient 592	24	2	1	1	0	0	#N/A	#N/A	#N/A
patient 593	26	2	2	2	0	0	#N/A	#N/A	#N/A
patient 594	20	2	2	1	0	0	#N/A	#N/A	#N/A
patient 595	25	2	1	1	0	0	#N/A	#N/A	#N/A
patient 596	27	1	1	2	0	0	#N/A	#N/A	#N/A
patient 597	23	2	1	1	0	0	#N/A	#N/A	#N/A
patient 598	39	1	2	1	0	0	#N/A	#N/A	#N/A
patient 599	26	3	1	1	0	0	#N/A	#N/A	#N/A
patient 600	22	2	1	2	0	0	#N/A	#N/A	#N/A
patient 601	29	2	1	2	0	0	185.5	5.6	7.78
patient 602	21	2	1	1	0	0	#N/A	#N/A	#N/A
patient 603	20	1	1	3	0	0	#N/A	#N/A	#N/A
patient 604	29	1	1	2	0	0	180.5	#N/A	4.64
patient 605	27	1	2	1	1	2	#N/A	#N/A	#N/A
patient 606	24	1	1	2	0	0	#N/A	#N/A	#N/A
patient 607	22	2	2	2	0	0	#N/A	#N/A	#N/A
patient 608	23	1	2	1	0	0	#N/A	#N/A	#N/A
patient 609	23	2	1	2	1	2	197.6	#N/A	#N/A
patient 610	28	1	2	1	0	0	#N/A	#N/A	#N/A
patient 611	30	1	2	1	0	0	#N/A	#N/A	#N/A
patient 612	24	1	1	1	1	2	#N/A	#N/A	#N/A
patient 613	32	1	3	1	1	2	#N/A	#N/A	#N/A
patient 614	31	2	1	1	0	0	#N/A	#N/A	#N/A
patient 615	26	1	1	1	1	3	#N/A	#N/A	#N/A
patient 616	27	2	2	2	0	0	#N/A	#N/A	#N/A
patient 617	24	2	1	1	0	0	#N/A	#N/A	#N/A
patient 618	28	1	2	1	0	0	#N/A	#N/A	#N/A
patient 619	25	2	1	2	1	2	#N/A	#N/A	#N/A
patient 620	25	3	1	1	0	0	#N/A	#N/A	#N/A
patient 621	29	1	2	2	0	0	#N/A	#N/A	#N/A
patient 622	28	1	2	1	1	3	#N/A	#N/A	#N/A
patient 623	22	2	1	2	0	0	#N/A	#N/A	#N/A
patient 624	25	2	1	1	0	0	144	#N/A	#N/A
patient 625	27	2	1	2	0	0	257.2	#N/A	4.39
patient 626	26	1	2	2	0	0	#N/A	#N/A	#N/A
patient 627	22	2	1	1	0	0	#N/A	#N/A	#N/A
patient 628	29	2	1	2	0	0	#N/A	#N/A	#N/A
patient 629	27	2	1	2	1	2	262.2	7.1	#N/A
patient 630	25	1	2	3	0	0	#N/A	#N/A	#N/A
patient 631	25	1	1	1	1	2	#N/A	#N/A	#N/A
patient 632	32	1	1	2	1	2	#N/A	#N/A	#N/A
patient 633	25	3	1	2	0	0	#N/A	#N/A	#N/A
patient 634	26	2	1	2	0	0	#N/A	#N/A	#N/A
patient 635	39	2	2	1	0	0	89.6	#N/A	3.33
patient 636	22	1	2	1	0	0	#N/A	#N/A	#N/A
patient 637	24	1	3	2	0	0	#N/A	#N/A	#N/A
patient 638	27	2	1	1	0	0	#N/A	#N/A	#N/A
patient 639	33	2	1	1	0	0	#N/A	#N/A	#N/A
patient 640	28	3	1	1	1	2	#N/A	#N/A	#N/A
patient 641	23	2	1	2	0	0	#N/A	#N/A	#N/A
patient 642	24	1	1	2	1	3	#N/A	#N/A	#N/A
patient 643	31	2	1	1	1	3	#N/A	#N/A	#N/A
patient 644	22	3	1	1	0	0	105.6	#N/A	3.97
patient 645	33	2	2	1	1	3	#N/A	#N/A	#N/A
patient 646	26	1	3	1	1	3	#N/A	#N/A	#N/A
patient 647	24	1	1	2	0	0	250.9	10.3	6.97
patient 648	25	1	1	1	0	0	#N/A	#N/A	#N/A
patient 649	28	2	2	2	0	0	268.5	10.9	#N/A
patient 650	26	1	3	1	0	0	#N/A	#N/A	#N/A
patient 651	19	1	1	2	0	0	282.8	#N/A	#N/A
patient 652	31	2	2	2	0	0	#N/A	#N/A	#N/A
patient 653	23	2	2	1	0	0	#N/A	#N/A	#N/A
patient 654	21	1	2	2	0	0	238	#N/A	6.33
patient 655	31	1	1	1	0	0	#N/A	#N/A	#N/A
patient 656	26	1	1	2	1	2	#N/A	#N/A	#N/A
patient 657	28	2	1	3	1	2	#N/A	#N/A	#N/A
patient 658	34	1	2	1	1	2	#N/A	#N/A	#N/A
patient 659	35	2	2	2	0	0	#N/A	#N/A	#N/A
patient 660	24	1	3	1	0	0	140.5	#N/A	4.13
patient 661	28	3	1	2	0	0	#N/A	#N/A	#N/A
patient 662	23	1	2	1	0	0	221.57	5.8	5.96
patient 663	31	1	1	2	0	0	200.65	#N/A	6.1

patient 664	26	3	1	2	0		0	#N/A	#N/A	#N/A
patient 665	29	3	1	1	0		0	#N/A	#N/A	#N/A
patient 666	28	2	1	1	0		0	#N/A	#N/A	#N/A
patient 667	23	2	1	1	0		0	#N/A	#N/A	#N/A
patient 668	32	1	2	1	1		2	#N/A	#N/A	#N/A
patient 669	27	3	1	2	0		0	#N/A	#N/A	#N/A
patient 670	23	1	2	1	0		0	#N/A	#N/A	#N/A
patient 671	37	2	1	1	0		0	#N/A	#N/A	#N/A
patient 672	21	1	1	1	0		0	#N/A	#N/A	#N/A
patient 673	26	1	2	1	0		0	#N/A	#N/A	#N/A
patient 674	29	3	1	1	0		0	124.9	#N/A	4.51
patient 675	25	1	1	1	1		2	#N/A	#N/A	#N/A
patient 676	25	1	2	3	0		0	#N/A	#N/A	#N/A
patient 677	32	2	1	2	1		2	#N/A	#N/A	#N/A
patient 678	29	3	1	2	0		0	151.9	#N/A	5.73
patient 679	39	3	1	1	0		0	232.73	#N/A	6.7
patient 680	26	1	1	2	0		0	#N/A	#N/A	#N/A
patient 681	33	1	2	1	0		0	#N/A	#N/A	#N/A
patient 682	27	2	1	2	1		2	90.7	#N/A	#N/A
patient 683	32	2	2	1	1		2	#N/A	#N/A	#N/A
patient 684	24	2	1	1	0		0	#N/A	#N/A	#N/A
patient 685	37	3	1	1	0		0	#N/A	#N/A	#N/A
patient 686	30	1	1	2	0		0	#N/A	#N/A	#N/A
patient 687	27	1	3	2	0		0	287.6	#N/A	#N/A
patient 688	22	2	2	3	0		0	#N/A	#N/A	#N/A
patient 689	21	1	3	1	0		0	#N/A	#N/A	#N/A
patient 690	27	2	1	2	1		2	#N/A	#N/A	#N/A
patient 691	30	1	2	1	0		0	#N/A	#N/A	#N/A
patient 692	23	2	1	3	0		0	#N/A	#N/A	#N/A
patient 693	22	1	3	1	1		3	#N/A	#N/A	#N/A
patient 694	22	2	1	2	1		2	#N/A	#N/A	#N/A
patient 695	24	1	1	2	0		0	#N/A	#N/A	#N/A
patient 696	24	2	1	2	0		0	#N/A	#N/A	#N/A
patient 697	23	1	2	2	1		2	#N/A	#N/A	#N/A
patient 698	30	1	2	1	0		0	191.3	#N/A	3.61
patient 699	31	1	1	1	1		2	#N/A	#N/A	#N/A
patient 700	30	1	1	1	0		0	#N/A	#N/A	#N/A
patient 701	25	1	1	2	0		0	181.4	#N/A	#N/A
patient 702	28	2	2	1	1		2	#N/A	#N/A	#N/A
patient 703	24	1	2	1	0		0	#N/A	#N/A	#N/A
patient 704	21	2	1	2	0		0	#N/A	#N/A	#N/A
patient 705	22	1	2	1	0		0	#N/A	#N/A	#N/A
patient 706	24	1	1	2	0		0	#N/A	#N/A	#N/A
patient 707	24	2	1	1	0		0	159.1	#N/A	5.07
patient 708	29	2	1	3	0		0	252.9	#N/A	#N/A
patient 709	26	2	1	2	0		0	#N/A	#N/A	#N/A
patient 710	24	1	2	1	0		0	#N/A	#N/A	#N/A
patient 711	37	1	1	1	0		0	#N/A	#N/A	#N/A
patient 712	37	3	1	1	1		4	#N/A	#N/A	#N/A
patient 713	34	1	1	2	1		2	#N/A	#N/A	#N/A
patient 714	20	1	1	1	0		0	#N/A	#N/A	#N/A
patient 715	39	2	1	1	0		0	#N/A	#N/A	#N/A
patient 716	23	1	1	1	0		0	199.5	#N/A	4.47
patient 717	24	1	2	1	0		0	#N/A	#N/A	#N/A
patient 718	35	1	2	3	0		0	115.8	#N/A	#N/A
patient 719	35	3	1	3	1		2	#N/A	#N/A	#N/A
patient 720	32	1	2	1	0		0	#N/A	#N/A	#N/A
patient 721	29	2	1	1	0		0	173.6	#N/A	4.63
patient 722	27	1	1	2	1		2	#N/A	#N/A	#N/A
patient 723	28	2	1	2	0		0	#N/A	#N/A	#N/A
patient 724	25	2	1	2	0		0	#N/A	#N/A	#N/A
patient 725	22	1	2	1	0		0	216.25	6.9	7.28
patient 726	27	1	2	2	0		0	215.83	#N/A	5.1
patient 727	28	3	1	3	0		0	163.8	#N/A	5.14
patient 728	22	1	1	1	0		0	207.7	5.2	6.88
patient 729	24	1	3	1	1		2	#N/A	#N/A	#N/A
patient 730	33	2	2	1	0		0	189.3	#N/A	#N/A
patient 731	32	1	1	2	0		0	211.65	6.2	4.12
patient 732	27	1	2	1	0		0	#N/A	#N/A	#N/A
patient 733	24	1	1	1	0		0	#N/A	#N/A	#N/A
patient 734	33	2	1	1	1	3		#N/A	#N/A	#N/A
patient 735	31	3	1	2	1		2	#N/A	#N/A	#N/A
patient 736	30	2	1	1	1	4		#N/A	#N/A	#N/A
patient 737	25	2	2	1	1		2	#N/A	#N/A	#N/A
patient 738	27	2	2	2	0		0	222.9	#N/A	4.47
patient 739	24	1	1	2	1		2	#N/A	#N/A	#N/A
patient 740	30	2	1	1	0		0	225.25	5.2	4.74
patient 741	26	1	2	2	0		0	194.9	#N/A	5.7
patient 742	24	2	2	1	0		0	278.05	6.25	4.23
patient 743	29	1	2	1	0		0	132.8	#N/A	5.93
patient 744	30	1	2	3	1		2	#N/A	#N/A	#N/A
patient 745	32	1	1	2	1		4	#N/A	#N/A	#N/A
patient 746	26	1	3	1	1	2		#N/A	#N/A	#N/A
patient 747	27	2	1	1	0		0	126.33	#N/A	6.31
patient 748	25	2	1	2	0		0	#N/A	#N/A	#N/A
patient 749	31	1	1	2	0		0	#N/A	#N/A	#N/A
patient 750	25	3	1	1	0		0	#N/A	#N/A	#N/A
patient 751	29	2	1	1	0		0	#N/A	#N/A	#N/A
patient 752	27	1	3	3	0		0	#N/A	#N/A	#N/A
patient 753	22	3	1	2	1		2	#N/A	#N/A	#N/A
patient 754	26	1	2	3	1		2	#N/A	#N/A	#N/A
patient 755	24	1	1	2	1		2	#N/A	#N/A	#N/A
patient 756	32	1	1	1	1		2	298.65	#N/A	#N/A
patient 757	35	2	2	1	1		2	#N/A	#N/A	#N/A
patient 758	29	2	1	1	1		3	#N/A	#N/A	#N/A

patient 759	17	1	2	3	0	0	#N/A	#N/A	#N/A
patient 760	34	1	1	1	1	2	250.1	#N/A	#N/A
patient 761	31	2	1	2	1	3	#N/A	#N/A	#N/A
patient 762	29	2	1	1	0	0	#N/A	#N/A	#N/A
patient 763	26	2	2	1	0	0	202.05	#N/A	#N/A
patient 764	28	1	3	2	1	2	#N/A	#N/A	#N/A
patient 765	31	1	2	2	1	2	#N/A	#N/A	#N/A
patient 766	25	1	2	2	1	2	#N/A	#N/A	#N/A
patient 767	26	1	2	3	1	3	#N/A	#N/A	#N/A
patient 768	23	2	2	2	0	0	#N/A	#N/A	#N/A
patient 769	24	1	1	1	1	2	#N/A	#N/A	#N/A
patient 770	29	2	2	1	1	2	#N/A	#N/A	#N/A
patient 771	25	2	1	1	1	3	#N/A	#N/A	#N/A
patient 772	28	1	1	1	0	0	248.05	5.4	4.93
patient 773	32	2	2	3	1	3	#N/A	#N/A	#N/A
patient 774	30	2	1	2	0	0	#N/A	#N/A	#N/A
patient 775	25	2	1	1	0	0	#N/A	#N/A	#N/A
patient 776	26	2	1	2	0	0	#N/A	#N/A	#N/A
patient 777	24	1	3	1	0	0	#N/A	#N/A	#N/A
patient 778	24	2	1	2	0	0	249.9	#N/A	6.14
patient 779	20	2	2	1	0	0	#N/A	#N/A	#N/A
patient 780	30	1	1	1	0	0	#N/A	#N/A	#N/A
patient 781	23	1	1	2	0	0	#N/A	#N/A	#N/A
patient 782	37	2	1	2	1	2	#N/A	#N/A	#N/A
patient 783	27	1	1	1	1	2	#N/A	#N/A	#N/A
patient 784	26	1	1	1	0	0	209.15	#N/A	5.92
patient 785	25	1	1	2	1	2	#N/A	#N/A	#N/A
patient 786	36	2	1	1	0	0	#N/A	#N/A	#N/A
patient 787	26	2	2	2	0	0	#N/A	#N/A	#N/A
patient 788	26	1	1	1	0	0	101.6	#N/A	4.34
patient 789	29	1	2	2	1	3	#N/A	#N/A	#N/A
patient 790	21	2	2	3	0	0	#N/A	#N/A	#N/A
patient 791	27	2	1	2	1	2	#N/A	#N/A	#N/A
patient 792	29	2	1	2	0	0	146.1	#N/A	4.65
patient 793	26	2	1	2	1	2	#N/A	#N/A	#N/A
patient 794	27	1	1	2	0	0	110.8	#N/A	#N/A
patient 795	26	1	2	1	0	0	#N/A	#N/A	#N/A
patient 796	29	1	2	2	0	0	299.3	#N/A	#N/A
patient 797	31	2	1	2	1	2	#N/A	#N/A	#N/A
patient 798	22	3	1	2	0	0	#N/A	#N/A	#N/A
patient 799	31	1	3	1	0	0	313.35	#N/A	5
patient 800	21	2	1	1	0	0	#N/A	#N/A	#N/A
patient 801	22	1	1	2	0	0	#N/A	#N/A	#N/A
patient 802	24	1	3	2	1	2	#N/A	#N/A	#N/A
patient 803	25	1	2	3	0	0	#N/A	#N/A	#N/A
patient 804	25	2	1	2	0	0	#N/A	#N/A	#N/A
patient 805	27	1	1	1	0	0	200.8	#N/A	4.42
patient 806	36	3	1	2	0	0	#N/A	#N/A	#N/A
patient 807	27	1	1	2	1	2	#N/A	#N/A	#N/A
patient 808	27	1	3	1	1	4	#N/A	#N/A	#N/A
patient 809	22	1	1	1	0	0	#N/A	#N/A	#N/A
patient 810	30	1	2	1	0	0	#N/A	#N/A	#N/A
patient 811	27	1	1	1	0	0	#N/A	#N/A	#N/A
patient 812	27	1	2	1	0	0	#N/A	#N/A	#N/A
patient 813	22	2	1	1	0	0	#N/A	#N/A	#N/A
patient 814	26	2	1	1	0	0	#N/A	#N/A	#N/A
patient 815	26	1	2	2	0	0	#N/A	#N/A	#N/A
patient 816	27	2	2	1	0	0	#N/A	#N/A	#N/A
patient 817	25	2	1	1	0	0	#N/A	#N/A	#N/A
patient 818	26	2	2	2	0	0	#N/A	#N/A	#N/A
patient 819	32	2	1	1	0	0	#N/A	#N/A	#N/A
patient 820	24	1	1	3	0	0	#N/A	#N/A	#N/A
patient 821	25	1	1	1	1	2	181.3	#N/A	#N/A
patient 822	30	1	2	1	1	2	#N/A	#N/A	#N/A
patient 823	31	2	2	2	0	0	#N/A	#N/A	#N/A
patient 824	25	2	1	1	0	0	#N/A	#N/A	#N/A
patient 825	30	2	1	1	0	0	#N/A	#N/A	#N/A
patient 826	25	1	1	1	1	2	#N/A	#N/A	#N/A
patient 827	26	2	1	1	0	0	#N/A	#N/A	#N/A
patient 828	24	2	1	2	1	3	#N/A	#N/A	#N/A
patient 829	22	1	1	1	0	0	#N/A	#N/A	#N/A
patient 830	28	1	1	2	1	2	#N/A	#N/A	#N/A
patient 831	24	1	2	1	0	0	201.3	#N/A	4.05
patient 832	24	2	1	1	0	0	#N/A	#N/A	#N/A
patient 833	21	2	1	1	1	2	#N/A	#N/A	#N/A
patient 834	24	1	2	1	0	0	#N/A	#N/A	#N/A
patient 835	23	1	2	2	1	2	#N/A	#N/A	#N/A
patient 836	27	2	2	2	1	2	#N/A	#N/A	#N/A
patient 837	25	2	2	2	1	2	#N/A	#N/A	#N/A
patient 838	20	2	1	2	0	0	#N/A	#N/A	#N/A
patient 839	28	1	2	1	0	0	#N/A	#N/A	#N/A
patient 840	27	1	2	2	0	0	120.1	#N/A	4.45
patient 841	25	1	1	2	0	0	126.8	#N/A	3.19
patient 842	20	1	2	1	0	0	#N/A	#N/A	#N/A
patient 843	22	2	1	2	0	0	#N/A	#N/A	#N/A
patient 844	22	2	1	1	0	0	#N/A	#N/A	#N/A
patient 845	22	2	1	1	0	0	#N/A	#N/A	#N/A
patient 846	20	1	3	1	1	2	216.3	#N/A	4.27
patient 847	26	1	1	3	0	0	#N/A	#N/A	#N/A
patient 848	32	1	2	2	0	0	#N/A	#N/A	#N/A
patient 849	30	1	1	2	0	0	#N/A	#N/A	#N/A
patient 850	42	1	1	2	0	0	#N/A	#N/A	#N/A
patient 851	28	2	2	1	0	0	#N/A	#N/A	#N/A
patient 852	26	2	1	2	0	0	#N/A	#N/A	#N/A
patient 853	32	1	2	1	0	0	#N/A	#N/A	#N/A

patient 854	22	1	2	2	1	2	#N/A	#N/A	#N/A
patient 855	38	1	3	1	0	0	#N/A	#N/A	#N/A
